# Three Cases of Resection of Bone

**Published:** 1851-10

**Authors:** Carter P. Johnson

**Affiliations:** Prof. of Anatomy and Physiology, Medical Department, Hampden Sydney College, Richmond, Va.


*Three cases of Resection of Bone.* By CARTER P. JOHNSON, M. D., Prof. of Anatomy and Physiology, Medical Department, Hampden Sydney College, Richmond, Va.

The following cases are reported, not because of any peculiar interest which attaches to any of them, but for the purpose of adding to the statistics with reference to an operation which has, perhaps, been too much neglected in this country.

*Case 1st. Resection of the Ulna.*—Rose, a negro girl, about 11 years of age, has been suffering for four years with a chronic inflammation of the right arm over the ulna. The disease was originally attributed to a fall or a blow which she received on the arm. The arm has occasionally suppurated, and from time to time, during the last year, several fragments of bone have been discharged.

When first presented to me, April 18th, 1849, there were two fistulous openings on the under side of the fore arm, and about two inches below the elbow joint, and the other about an inch lower down, through which the probe passed freely down to the denuded ulna. Neither the elbow joint nor the radio-ulnar joint below, seemed to be involved in the disease. The general health of the child had now begun to suffer, and it was evident that she could not long stand the continued irritation to which she had already so long been subjected.

On April 21st I exsected the middle two-thirds of the diseased bone, the patient being placed fully under the influence of ether. The operation was performed by making two semi-elliptical incisions, extending from a point a few lines below the internal condyles to the styloid process of the ulna, meeting at their extremities, and including between them the fistulous orifices and the diseased integument; then dissecting away the integuments and muscles from the anterior and posterior surfaces of the ulna, dividing this bone just above its styloid process with the metacarpal saw, separating its attachment to the interosseous membrane, and completed by making the upper section of the bone with the chain saw, just below the coronoid process. But little blood was lost, and only one vessel required the ligature. The edges of the wound were then brought together by adhesive strips, and an angular splint applied, extending beyond the ends of the fingers.

With the exception of fever, accompanied with slight delirium, on the second day after the operation, which was speedily checked by a mercurial cathartic, the patient recovered rapidly, and was discharged about eight weeks after the operation, entirely well.

By adopting the operation of exsection in this case, the elbow and the wrist joints were both saved to this patient; and before her discharge she could use them both freely. Before the introduction of the operation of exsection, this arm would have been amputated, probably above the elbow.

*Case 2d. Resection of two-thirds of inferior maxilla.*—In July, 1850, I was asked to see Mary, a negro woman, aged about 35 years. She gives the following history of her case. About twelve months ago, while in New Orleans, she had the first molar tooth of the left side of the lower jaw extracted: she states that in the operation a portion of the alveolar process was broken off, and that from that time she has suffered constant pain and swelling in that side of the face, with occasional discharge of small pieces of bone.

When I saw her, the left side of the face was much swollen, and the whole region of the left side of the inferior maxillary bone the seat of much induration; there was a fistulous orifice externally along the lower margin of the bone, about half way between the angle and the symphysis, which communicated with the diseased bone, and another orifice which opened internally on the gum, whence the molar tooth had been extracted, which also communicated with the denuded bone and with the other sinus.

On the 12th of July, hoping to be able, by a simple operation, to remove the whole cause of the trouble, I made an incision over the seat of the disease, and finding that a large portion of the thickness of the bone was involved, took out an entire section, of a V shape; after the external wound resulting from this operation was entirely healed, as it was in three weeks, there still remained a fistulous orifice in the mouth, which was found to communicate with denuded bone; from this orifice several pieces of necrosed bone, were from time to time extracted, without, however, affording any permanent relief to the patient, whose face continued much swollen and very painful. In a few days, an external orifice again made its appearance, from which a considerable quantity of pus was discharged daily, and which was found also to communicate with denuded bone.

Upon a careful examination of the case now, it was ascertained that the whole left side of the body of the bone was in a state of hypertrophy, the disease extending across the symphysis to a point between the right lateral incisor and the canine tooth of the same side in one direction, and to the angle of the bone in the other.

Upon consultation with my friends, Drs. Marx and Gibson, it was determined that the safety of the patient depended upon the entire removal of the disease, and that to effect this with greater certainty, it would be better to disarticulate the bone at the temporo-maxillary articulation, and to resect the body just to the right of the diseased portion.

Accordingly, on the 2nd of September, I proceeded to do the operation as follows:—The patient being placed in a suitable chair, and put thoroughly under the influence of chloroform, an incision was made commencing opposite the left tempero-maxillary joint, passing downwards along the ramus, a few lines below the angle, thence, slightly curved with the convexity downwards, horizontally along the inferior edge of the body of the bone, to the right side of the chin, and thence upwards to the right angle of the lip. The dissection was then made from below upwards, so as to penetrate the cavity of the mouth, and to expose the whole of the anterior face of the diseased mass. A slight dissection and the use of the fingers enabled me to detach the soft parts from the posterior face of the bone along the portion at which the section was to be made. This accomplished, the first incision was extended through the lower lip,* the flap turned back and held by an assistant, the chain saw passed behind the bone just anterior to the first bicuspid tooth, and the bone rapidly divided at that point. The internal attachments of the bone were then speedily divided, and the operation completed by the division of the attachment of the temporal muscle to the coronoid process and the disarticulation at the glenoid cavity. This stage of the operation was greatly facilitated by using the bone itself as a lever, to put upon the stretch the parts to be divided and to pry out the coronoid process from under the zygoma.

No vessel required ligaturing. The edges of the coronoid were brought together with sutures and adhesive strips, a light dressing applied and the patient put to bed, after the administration of a stimulant, in a tolerably comfortable condition.

During the operation, a thread was passed through the arteries of the frenum of the tongue, in case retraction should take place ; but although the bone was divided some distance to the right of the symphysis, and consequently the anterior portion of the genio-hyo-glossus muscle necessarily divided, no retraction of the tongue took place, and no symptoms of suffocation occurred.

Upon an examination of the bone after the removal, it was found in a state of hypertrophy, the cancellated portion and the

*Anxious to preserve the integrity of the lower lip, if possible, I attempted to pass the saw without dividing it, but found that the extreme thickness of the bone and the small curve of the needle used, rendered it impossible. I was obliged, therefore, to divide the lip in order to obtain more space for manipulation.

dental canal in the left side being filled up with dense compact laminated bone structure. Though but seven weeks since the first operation, the space left by the removal of the section of bone at that time had become firmly filled with cartilaginous tissue.

The patient did well and on the 17th September, just two weeks after the operation, the wound was completely healed. There remained, however, partial paralysis of the left side of the face owing to the section of the portio-dura, and a good deal of swelling, with some eversion of the lower lip. The condition of the lip was entirely relieved in the course of a few weeks longer by the use of adhesive strips, and the paralysis gradually diminished, though it has not entirely disappeared.

*Case 3rd. Resection of the ununited ends of a fractured Femur.*—The history of this case having been already published in the Stethoscope for May, I will merely describe the operation and its result.

The patient, an athletic Irishman, 19 years of age, had suffered just three months and ten days before, a very oblique fracture in the upper third of the right thigh, in a direction from above downwards and forwards; the fragments were consequently, at the time of the operation, very far separated from each other, the lower fragments being drawn upwards and inwards.

Having placed the patient fully under the influence of chloroform, the operation was commenced by an incision extending from the trochanter major to the outer surface of the external condyle. This incision extended down to the fascia lata, which was then divided continuously with the first incision. In order now to reach the lower end of the upper fragment of the fractured bone, which was drawn forwards and outwards, the vastus externus was separated from its attachments to the rectus femoris, and held back by an assistant. With some little difficulty the extremity sought for was found, a chain saw passed around it about an inch from its termination, the section readily made, and the fragment separated from its ligamentous attachments and removed.

The next step in the proceeding, in the section of the upper extremity of the lower fragment, proved very difficult, in consequence of the great retraction of this portion of the fractured bone. This lower fragment was found drawn up to the lower portion of the gluteal region, and carried by the adductors very considerably inwards, rendering its access by an external incision very difficult. Finding it impossible to reach the upper portion of this fragment through the separation already made between the vastus and the rectus, the vastus was separated from its posterior attachments and turned forward. After a tedious dissection, in which the handle of the scalpel and the fingers were used very much to the exclusion of any cutting instrument, in order to avoid the risk of dangerous hemorrhage, the extremity of the bone was at length reached, and with some difficulty, owing to its very great depth, the chain saw passed around it. This portion of the operation was much facilitated by carrying the foot across the opposite instep, so as to force the fragment sought nearer to the external surface of the thigh. The chain saw turned down both in front and behind, by the thick muscles of the thigh, was worked with difficulty, and, before the section was completed, broke. A good metacarpal saw which was at hand, was substituted, and with it the section finished. The detached portion about an inch and a half in length, was then easily removed. The two opposite ends of the bone, thus rendered perfectly transverse, could then be brought accurately into contact, producing a shortening of the limb of about two and a half inches.

The next step in the operation consisted in boring with a small bit and brace, a hole in each of the ends of the bone thus approximated, through which a very strong silk ligature was passed by means of an eye-probe and finally tied. The holes were bored obliquely, commencing each about half an inch from the sawn extremity, and passing the one upwards and the other downwards, terminated in the medullary canal. The ends of the ligature were brought out of the wound. The edges of the wound were then brought together as accurately as possible, the limb lightly bandaged, a long splint applied along the whole posterior surface of the limb, and the patient put to bed, with the leg resting upon a slightly inclined plane.

During the operation, a good deal of blood was lost, a large portion of which was venous; and after its completion, the patient who had then recovered from the effect of the anæsthetic agent, was very pallid and excessively prostrated. Brandy and water was freely administered, and warm clothing applied. Reaction took place very slowly; about four hours after the operation, the pulse began to rise and the skin to regain its warmth. The stimulants were then used more moderately. The reaction, however, was never complete, and only lasted a few hours. During the night the pulse again began to flag, and the patient became more restless; stimulants were again resorted to and used very freely, but with no effect. The system seemed to have lost its power of responding to any agent, and in spite of the most active stimulant treatment, the patient continued gradually to sink, until about 6 o'clock the next evening, just thirty hours after the operation, he died.

No post-mortem examination could be obtained.

